# Leftward asymmetry of the planum temporale and its association with language

**DOI:** 10.1007/s00429-025-02980-y

**Published:** 2025-07-04

**Authors:** Emma M. Karlsson, Sebastian Ocklenburg

**Affiliations:** 1 Department of Experimental Clinical and Health Psychology, Ghent University, Ghent, Belgium; 2 Department of Psychology, MSH Medical School Hamburg, Hamburg, Germany; 3 ICAN Institute for Cognitive and Affective Neuroscience, MSH Medical School Hamburg, Am Kaiserkai 1, 20457 Hamburg, Germany; 4 Biopsychology, Institute of Cognitive Neuroscience, Faculty of Psychology, Ruhr University Bochum, Bochum, Germany

**Keywords:** Laterality, Lateralization, Hemispheric asymmetry, Brain asymmetry, Language, Handedness

## Abstract

Several cortical brain regions show structural left-right asymmetries. One of the most pronounced forms of structural asymmetry in the human brain is the leftward macrostructural asymmetry of the planum temporale, the posterior part of the superior surface of the temporal lobe. The planum temporale overlaps with Wernicke’s area, a core structure in the brain network involved in sensory language processing. Therefore, several studies have investigated the association between macrostructural leftward asymmetries of the planum temporale and functional leftward asymmetries in language processing. However, the results of these studies have been ambiguous and sometimes contradictory. In this mini-review article, we argue that asymmetric structure-function associations in the language system cannot be fully understood by only examining averaged asymmetries obtained from macrostructural measures such as volume, cortical thickness, or surface area. Recent in-vivo neuroimaging studies, along with earlier post-mortem histological studies, suggest that the planum temporale also shows substantial leftward asymmetries in its microstructural organization. These microstructural asymmetries concern the columnar organization of the planum temporale and the density of neurites. Importantly, recent studies have shown that microstructural asymmetries in the planum temporale exhibit stronger associations with functional hemispheric lateralization of the language system than macrostructural ones. Based on these findings, we suggest that the association between structural and functional asymmetries in the language system can only be understood if macrostructural and microstructural asymmetries are both considered.

## Introduction

**Fig. 1:**
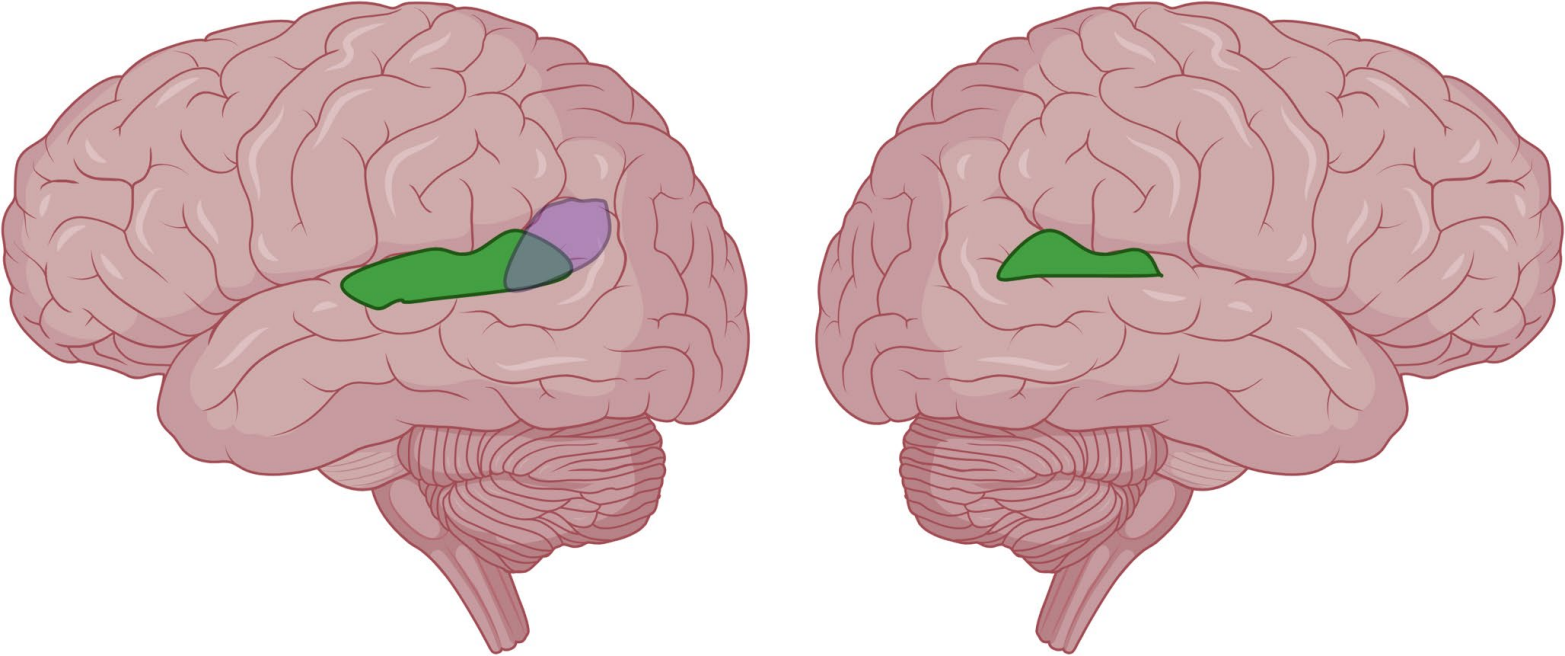
Schematic illustration of the leftward macrostructural asymmetry of the planum temporale (green) and its approximate anatomical relationship to Wernicke’s area (purple) in the left hemisphere. Figure created in BioRender. Ocklenburg, S. (2025) https://BioRender.com/ mzpxrpi

The human brain shows a large number of structural left-right differences, also known as hemispheric asymmetries (Guadalupe et al. 2017; Kavaklioglu et al. 2017; Kong et al. 2018, 2022; Kuo and Massoud 2022). According to a commonly used categorization scheme (Amunts 2010), structural hemispheric asymmetries are observed at multiple levels (Güntürkün et al. 2020): the macroscopic level (e.g., volume, surface area, or cortical thickness of brain areas) (Sha et al. 2021), the microstructural level (e.g., number and density of axons and dendrites in a brain area) (Wan et al. 2024), and the molecular level (e.g., asymmetries in gene expression) (Schijven et al. 2024). A large-scale study by the ENIGMA consortium showed that 76.5% of cortical brain areas show significant asymmetries in cortical thickness, and 91.1% show significant asymmetries in surface area (Kong et al. 2018). Among cortical areas, one of the most pronounced forms of structural asymmetry is the leftward macrostructural asymmetry of the planum temporale (PT), the posterior part of the superior surface of the temporal lobe (see Fig. 1) (Steinmetz 1996; Beaton 1997; Shapleske et al. 1999; Kuo and Massoud 2022). The PT overlap with Wernicke’s area, a core structure in the brain network involved in sensory language processing, making it a key region of interest for many neuroimaging studies on language.

### The aim of the present review Article

The neuroanatomical details of macrostructural asymmetries in the PT have been described in detail in several excellent review articles from the 1990s (Steinmetz 1996; Beaton 1997; Shapleske et al. 1999). More recently, review articles and book chapters (Amunts 2010; Kuo and Massoud 2022) have discussed PT asymmetries within the broader context of structural asymmetries. Rather than reiterating the broader literature, we briefly summarize key findings and focus specifically on the association between PT asymmetries and functional language lateralization. Specifically, we highlight recent findings suggesting that understanding the association between PT asymmetries and functional language lateralization requires focusing beyond macrostructural asymmetries. It is crucial to also take microstructural PT asymmetries into account.

### Macrostructural asymmetries in the PT

Macrostructural asymmetry in the PT rose to prominence with a seminal paper by Geschwind and Levitsky in 1968, who reported that the left PT was larger in 65% of brains, compared to only 11% in which the right PT was larger (Geschwind and Levitsky 1968). This overall leftward asymmetry in the volume of the PT has been replicated many times (Steinmetz 1996; Beaton 1997; Shapleske et al. 1999; Toga and Thompson 2003; Tzourio-Mazoyer et al. 2010, 2018; Tzourio-Mazoyer and Mazoyer 2017; Kopal et al. 2024), and reproduced in recent large-scale studies. For example, a neuroanatomical study of 2337 individuals from the general population (Guadalupe et al. 2015) reported that, on average, males had a left PT volume of 2035 mm^3^ (SD = 278) and a right PT volume of 1543 mm^3^ (SD = 208). Females had a left PT average volume of 1807 mm^3^ (SD = 242) and a right PT average volume of 1406 mm^3^ (SD = 178). Similarly, a recent analysis of 18,057 individuals from the UK Biobank also reported a significant group-level leftward asymmetry of PT grey matter volume (Carrion-Castillo et al. 2020). Since handedness also shows a strong asymmetry (Ocklenburg et al. 2025; Ocklenburg and Güntürkün 2025) and it has been suggested that it may be associated with macrostructural brain asymmetries (Sha et al. 2021), several studies have investigated the association between handedness and macrostructural PT asymmetries. However, handedness does not seem to have a strong effect on PT asymmetries. Two large studies did not observe handedness-related differences in surface-based asymmetry of the superior temporal lobe, despite including over 100 and 3,000 left-handers, respectively (Guadalupe et al. 2014; Sha et al. 2021). Similarly, an analysis of 99 datasets reported no consistent effects, aside from an uncorrected difference in one sample (Kong et al. 2018). In contrast, an older study reported reduced leftward PT asymmetry among people who were classified as left-handed according to motor tasks (Steinmetz et al. 1991). These discrepancies most likely reflect methodological variation, including differences in sample size, imaging protocols, or anatomical definitions of the PT.

### Microstructural asymmetries in the PT

In addition to macrostructural asymmetries in the PT, asymmetries have also been reported on the microstructural level. Post-mortem studies have indicated that the human temporal cortex, including the PT, shows a leftward asymmetry in modular structure (Seldon 1981a, b, 1982; Galuske et al. 2000). Specifically, it was found that the left PT contains more functionally distinct neural microcolumns per surface unit than the right PT (Galuske et al. 2000; Hutsler and Galuske 2003). A comparative study of post-mortem brains from humans, chimpanzees, and Rhesus monkeys reported that the human brain had a robust asymmetry with wider neuronal minicolumns and more neuropil space in the left hemisphere (Buxhoeveden et al. 2001). This microstructural PT asymmetry was absent in the non-human primates. Moreover, the language cortex of the left hemisphere contains a greater number of the largest pyramidal cells compared to the right hemisphere (Hutsler 2003), indicating a microstructural asymmetry in this specific type of neuron.

**Fig. 2:**
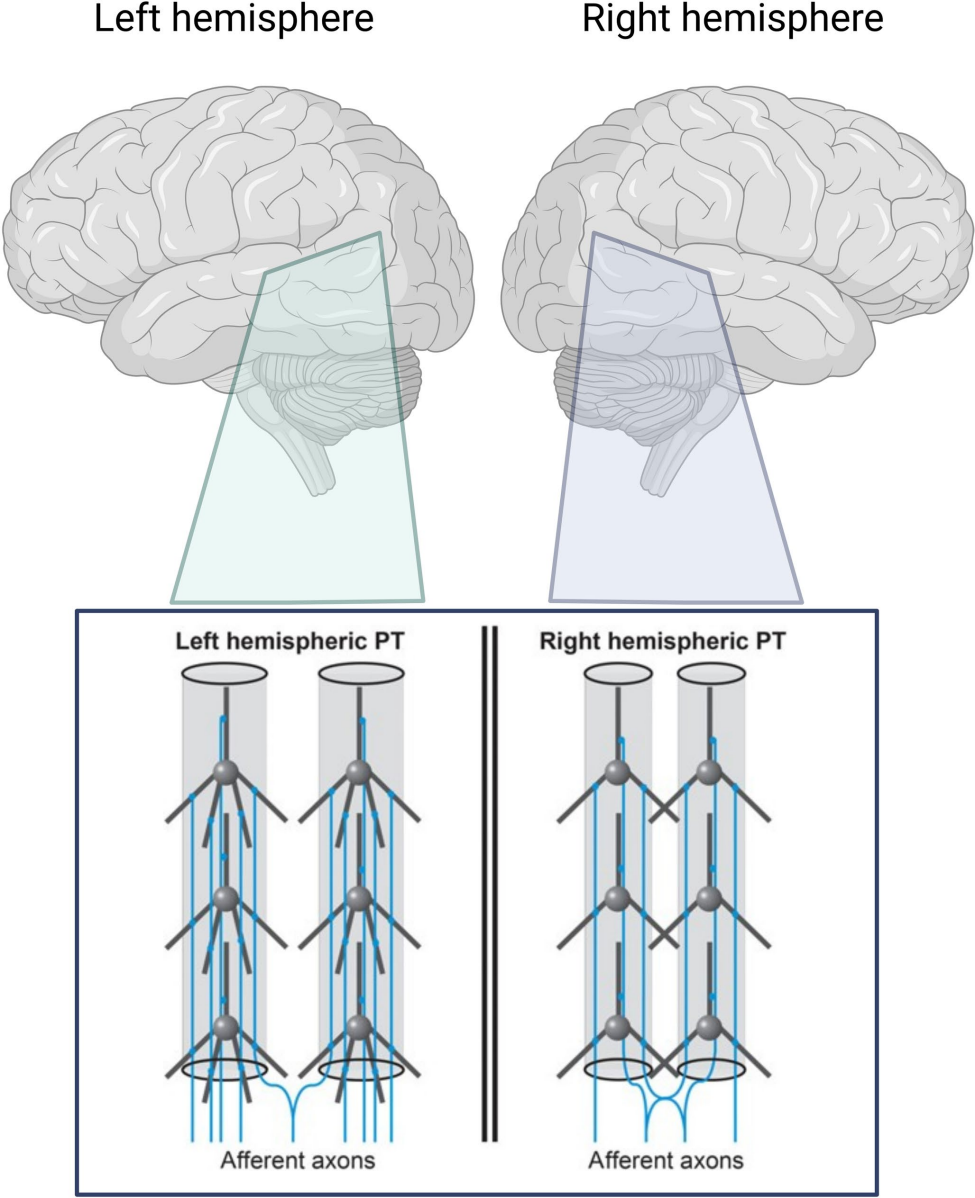
Schematic figure showing microstructural asymmetries in the PT (Ocklenburg et al. 2018). The left PT shows a higher neurite density than the right PT, with more dendrites being present in the left hemisphere. Figure created in BioRender. Ocklenburg, S. (2024) https://BioRender.com/h21g266. Upper panel original content created in Biorender. Lower panel from a previously published paper by one of the co-authors of this paper, published under a Creative Commons Attribution NonCommercial License 4.0 (CC BY-NC) (Ocklenburg et al. 2018)

Building on these post-mortem findings, recent in-vivo studies using NODDI (neurite orientation dispersion and density imaging) (Jespersen et al. 2010, 2012; Zhang et al. 2012; Ocklenburg et al. 2018) have also investigated microstructural asymmetries of the brain (Schmitz et al. 2019a, b; Mundorf et al. 2021b), and the PT more specifically (Ocklenburg et al. 2018). Here, it was found that the NODDI intra-neurite volume fraction, a marker of neurite density, was significantly higher in the left compared to the right PT (Ocklenburg et al. 2018). This suggests a higher concentration of axons and dendrite in the left PT compared to the right (see Fig. 2). Moreover, the NODDI orientation dispersion index also showed a significant leftward bias, indicating stronger dendritic arborization in the left hemisphere.

More recently, a study based on data from 907 participants also found a significant leftward asymmetry in the NODDI orientation dispersion index (Qin et al. 2024). Interestingly, neurite density in the PT was dependent on the structure of Heschl’s gyrus (HG). Unlike previous studies, the study by Qin et al. (2024) analyzed PT asymmetries in the context of whether participants had a duplication of HG, the brain area superior to the PT. Individuals with one HG in the left hemisphere and two HGs in the right hemisphere showed a significant leftward asymmetry of neurite density in the PT. However, individuals with only one HG in the right hemisphere showed a significant rightward asymmetry of the PT, and individuals with a duplication of HG in both hemispheres showed no significant PT asymmetries. This shows that the individual HG duplication is a critical factor for macrostructural PT asymmetries and should be considered when analyzing them and linking them to behavioral markers. Not doing so may induce a confounding factor and mask potentially relevant results in specific subgroups with different HG patterns.

A study assessing microstructural asymmetries in intracortical myelination (also known as T1/T2 ratio) in the PT reported complementary findings to the NODDI results (Tzourio-Mazoyer et al. 2019). In this study, the authors tested 445 participants and manually delineated the PT and the Heschl gyrus in the MRI images. They found a significant leftward asymmetry of the PT for intracortical myelination.

### Molecular asymmetries in the PT

Molecular asymmetries in the PT have not been a major focus of research on hemispheric differences in the human brain. However, a recent genome-wide association study (GWAS) on genetic effects on PT asymmetries utilizing the UK Biobank also reported data on mRNA expression analysis in post-mortem PT tissue in the Allen Brain Atlas microarray data (Carrion-Castillo et al. 2020). The GWAS identified two loci (rs41298373 and rs7420166) that were significantly associated with PT asymmetry. Interestingly, the rs7420166 locus is known to affect the expression of the genes *BOK* and *DTYMK*. Importantly, the analysis of the Allen Brain Atlas microarray data indicated that *DTYMK* showed significant leftward asymmetry of mRNA expression in post-mortem PT tissue. This finding suggests that molecular asymmetries in gene expression are likely present in the PT. However, more systematic research on molecular asymmetries in the PT is needed.

### Asymmetries of the planum temporale in non-human animals

Macrostructural PT asymmetries are not unique to the human brain. Recent studies have shown that several non-human primate species also have these asymmetries. For example, a study found that the left PT was significantly larger in 17 out of 18 (94%) chimpanzees (Gannon et al. 1998). A larger, more recent study also reported population-level leftward asymmetries for PT grey matter volume and surface area in chimpanzees (Hopkins and Nir 2010). A study comparing several great ape species to lesser apes and Old World Monkeys found significant leftward asymmetry of the PT in great apes, but not in the other primate species, in which the PT could not be identified (Hopkins et al. 1998). Another study involving four great ape species also reported a larger PT in the left hemisphere than in the right (Cantalupo et al. 2003). However, macrostructural PT asymmetries are not limited to great apes. A study of 96 Olive Baboons showed a clear leftward asymmetry of the PT (Marie et al. 2018), and a similar result was found in newborn Olive Baboons (Becker et al. 2021). Taken together, these studies show that macrostructural asymmetry of the PT is not unique to the human brain. Since these primate species lack language, some have suggested that the PT asymmetry in these species may be relevant for gestural communication (Becker and Meguerditchian 2022). This idea has recently been supported by a study showing that PT asymmetry in newborn monkeys predicts the later development of handedness for gestural communication (Becker et al. 2024).

## Language lateralization

**Fig. 3:**
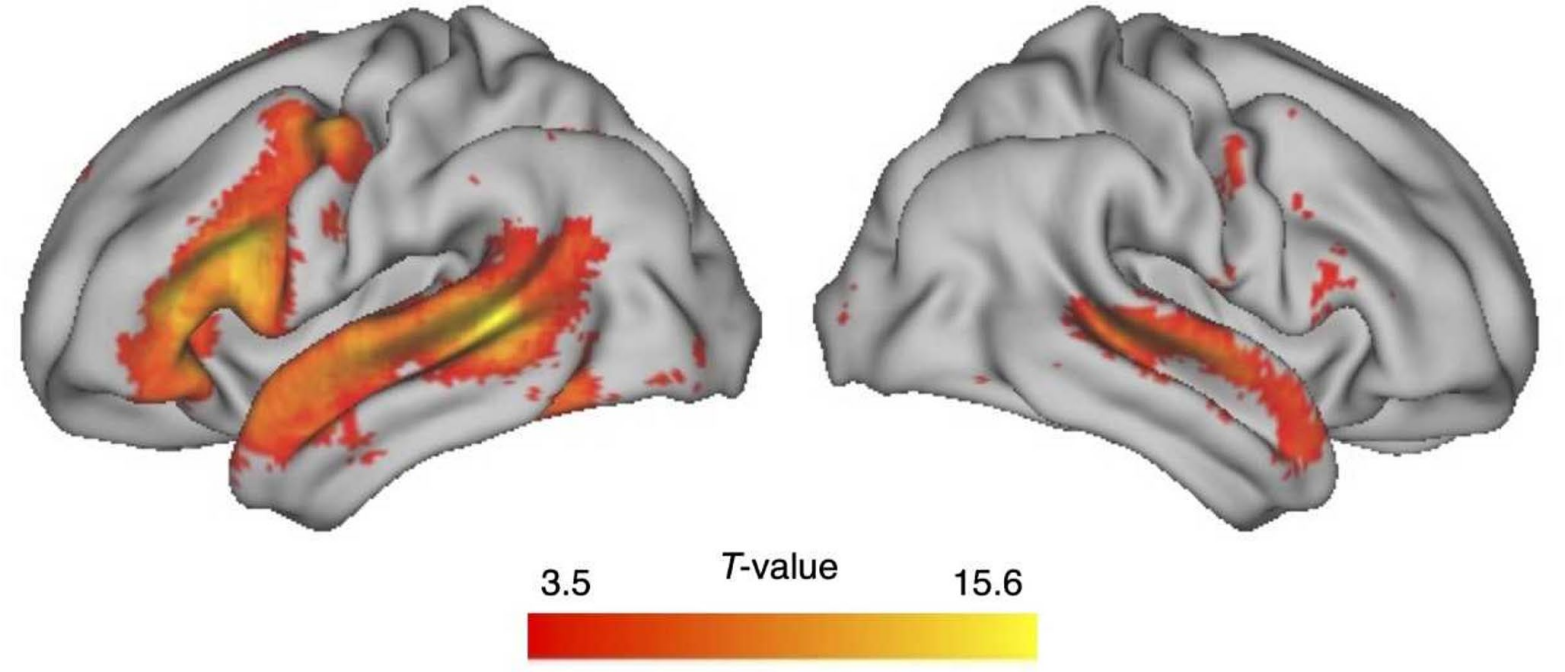
Functional activation using the term “language” in Neurosynth (Yarkoni et al. 2011) created from a meta-analysis of 1101 studies visualized using Connectome Workbench (Marcus et al. 2013). The left panel shows the lateral surface of the left hemisphere and the right panel the lateral surface of the right hemisphere. Left lateralization elicited by language studies is clearly demonstrated

One primary reason why the structural asymmetry of the planum temporale is of interest in cognitive neuroscience is its overlap with Wernicke’s area, a core structure in the brain network responsible for sensory language processing (see Fig. 1). Several functional language-related processes, including auditory language processing (Tervaniemi and Hugdahl 2003), language production (Mazoyer et al. 2014) and reading (Price and Devlin 2011) are predominantly left-lateralized in the majority of individuals (Fig. 3).

The discovery of the left hemisphere’s dominant role in language processing can be traced back to the pioneering work of Paul Broca and Marc Dax in the early to mid-1800s (Friedrich et al. 2019), who linked speech production disturbances with damage to the left inferior frontal gyrus. Broca’s findings were pivotal in establishing the important connection between speech and the left cerebral hemisphere (Keller et al. 2009). Since then, this approximate region has become known as Broca’s area, now typically defined as the pars opercularis and pars triangularis of the inferior frontal gyrus (IFG). Later, Carl Wernicke identified the left posterior superior temporal gyrus as crucial for language comprehension, now referred to as Wernicke’s area (Geschwind 1967).

Large-scale, systematic investigations of language lateralization advanced with the development of the Wada test (Wada 2008). This procedure involves injecting sodium amytal into the left or right internal carotid artery, temporarily anesthetizing the corresponding hemisphere while the patient performs speech tasks (Branch et al. 1964). These studies provided insights into the incidence of left hemisphere dominance for language. For instance, Rasmussen and Milner (1977) examined 140 right-handed and 122 non-right-handed epilepsy patients without evidence of early left-hemisphere injury (Rasmussen and Milner 1977). Among right-handers, 96% were left-hemisphere dominant, and 4% were right-hemisphere dominant. These rates were reduced among non-right-handers, where 70% were left hemisphere dominant, 15% were right hemisphere dominant, and 15% showed bilateral dominance (i.e., speech was either unaffected or lost regardless of which hemisphere was anesthetized). A meta-analysis further confirmed a 15–25% reduction in left-sided language dominance among non-right-handers compared to right-handers, based on various techniques for determining language lateralization, including Wada, electroconvulsive therapy, transcranial magnetic stimulation, behavioral methods, and neuroimaging (Carey and Johnstone 2014).

Given the invasive nature of the Wada test, behavioral and neuroimaging techniques are now the most widely used methods for assessing language lateralization in both healthy and clinical populations. Dichotic listening (DL) is a common behavioral technique where auditory stimuli are presented simultaneously to both ears (Westerhausen and Hugdahl 2008; Hugdahl et al. 2009; Hugdahl and Westerhausen 2016). A right-ear advantage in word perception is typically interpreted as evidence of left-hemisphere language dominance. A meta-analysis of 67 studies with right-handers and 57 studies with left-handers showed that DL revealed a right ear advantage in 81% of right-handers and 67% of left-handers (Karlsson et al. 2019).

Behavioral methods, however, provide only indirect evidence of language lateralization. In contrast, functional neuroimaging techniques offer a more direct means of assessing neural asymmetries. One such method is functional transcranial Doppler ultrasound (fTCD), which measures changes in cerebral blood flow velocity in the middle cerebral arteries during task performance (Badcock and Groen 2017). The most commonly used task to measure language lateralization with fTCD is the word generation task, where participants covertly or overtly generate words while blood flow velocity is recorded. A recent study of 103 right-handers and 110 left-handers found that 75% of left-handers and 86% of right-handers were left-lateralized during the verbal fluency task (Parker et al. 2022). Interestingly, fTCD studies that examined lateralization across multiple language tasks in the same participants found task-dependent variability in lateralization (Woodhead et al. 2021; Parker et al. 2022). Left-handers, in particular, showed greater variability in the hemisphere lateralized for specific tasks. Thus, the extent of left-lateralized language asymmetry can also vary depending on the language task used.

Functional MRI (fMRI) studies have similarly most often relied on verbal or sentence fluency tasks to measure language lateralization (Häberling et al. 2016; Johnstone et al. 2020; Kroliczak et al. 2021). For example, a sentence generation task involving 144 right-handers and 153 left-handers found that 94% of right-handers and 84% of left-handers were left-lateralized for language, based on a left/right classification of the functional activation (Mazoyer et al. 2014). In a recent large-scale study, the authors (Malik-Moraleda et al. 2022) examined language lateralization across 45 different languages using an auditory language task in the participants’ native languages. They found that the different languages all activated a fronto-temporo-parietal network that showed a leftward asymmetry, suggesting a universal neural architecture for language that is robust across linguistic diversity. Taken together, the converging evidence from different research methods shows that most individuals (about 81–96% of right-handers and 67–84% of left-handers) are left-lateralized for language processing, although the extend of the lateralization can vary depending on the specific language task or region(s) that are examined (for a review of fMRI language lateralization studies, see (Bradshaw et al. 2017).

## The association between structural asymmetries in the planum temporale and functional Language lateralization

As outlined in the previous section, language processing show a pronounced functional leftward asymmetry in most individuals (Ocklenburg et al. 2014; Bless et al. 2015; Malik-Moraleda et al. 2022; Karlsson et al. 2023). Given the pronounced leftward structural asymmetry of the PT and its overlap with a core region of the language processing network, it comes as no surprise that considerable effort has been made to investigate whether PT asymmetries predict functional language lateralization. Such investigations can help us better understand why asymmetrical structures and functions arise in the brain and how they support language processing. In addition, this line of research may also have clinical relevance for conditions such as epilepsy, stroke, and developmental language disorders, where typical patterns of language lateralization may be disrupted. Improved knowledge of the structure–function relationships may support more targeted diagnostic and therapeutic approaches. Most of these studies so far have focused on macrostructural asymmetries, such as PT volume, but their findings remain inconsistent and sometimes contradictory.

Some studies report an association between macrostructural PT asymmetries and functional language lateralization. For example, a study that combined magnetic resonance imaging of the PT with the Wada test to determine language dominance found that 11 participants with leftward functional language dominance also had a leftward asymmetry of the PT (Foundas et al. 1994). One participant with strong rightward asymmetry of the PT also showed rightward language lateralization. However, other studies failed to identify any associations. For example, a study with 52 participants did not find any significant associations between functional laterality indices from four different verbal dichotic listening tests and macrostructural PT asymmetry (Jäncke and Steinmetz 1993). Similarly, a more recent study in *N* = 287 participants reported no relationships between PT asymmetry and functional asymmetries during two different language tasks: one involving production and one involving perception (Tzourio-Mazoyer et al. 2018).

Greve et al. examined PT asymmetry in left-handers, comparing those who were right-lateralized (*n* = 21) to those who were left-lateralized (*n* = 32) during a language production task. (Greve et al. 2013). They found significant leftward PT asymmetry in both groups, suggesting no clear association between structural and functional asymmetries. However, their whole-brain surface-based analysis did identify a significant cluster within the PT that was associated with language lateralization, suggesting that macrostructural asymmetries may still play a role, albeit at a more spatially localized level than PT as a whole. Some studies also found effects only in subgroups of participants. For example, Dos Santos Sequeira et al. (2006) found a significant association between PT asymmetries and language lateralization determined with dichotic listening only in right-handed males, but not in left-handed males or females of any handedness (Dos Santos Sequeira et al. 2006). Overall, the results of studies relating macrostructural PT asymmetry to functional language lateralization are largely ambiguous and sometimes contradictory. Thus, there is presently no convincing evidence linking macrostructural asymmetry to language asymmetry, making it clear that other structural properties of the brain may be more relevant in this context.

Interestingly, it has been argued that lateralized cognition has a microstructural, not macrostructural basis (Chance 2014). Chance (2014) suggested that language dominance is based on cortical microcolumn asymmetries. A recent study using NODDI also supported this idea (Ocklenburg et al. 2018). Ocklenburg et al. (2018) investigated both macrostructural and microstructural PT asymmetries and used electroencephalography to assess functional language lateralization in the same individuals. While there was no association between PT volume asymmetry and functional language lateralization, neurite density in the PT significantly predicted the latency of the EEG N1 ERP component to verbal stimuli in the same hemisphere. The N1 typically shows a leftward lateralization at posterior electrode sites in response to verbal stimuli (Grossi et al. 2010) which is why it is used as an EEG marker of language lateralization.

Similarly, Qin et al. (2024) assessed the relationship between functional language lateralization for speech comprehension and perception assessed with fMRI, and PT asymmetries in several macrostructural and microstructural measures, including surface area, cortical thickness, myelin content, neurite density, and orientation dispersion (Qin et al. 2024). For speech perception, they found significant positive correlations with asymmetries in myelin content, neurite density, and orientation dispersion, but not for the macrostructural measures of surface area and cortical thickness asymmetries. For speech comprehension, asymmetries in surface area, myelin content, and neurite density significantly correlated with functional lateralization. All significant correlations were positive, suggesting that stronger structural PT asymmetries were associated with stronger functional language lateralization.

For the above mentioned study on intracortical myelination, structural PT asymmetries were correlated with functional hemispheric asymmetries in behavioral language tasks (Tzourio-Mazoyer et al. 2019). Rhyming performance decreased with an increase of the size of the right PT. Taken together, these findings suggest that it is crucial to look beyond macrostructural PT asymmetries when investigating the structural correlates of functional language lateralization. To gain a deeper understanding if structural asymmetries drive functional language lateralization, it is essential to consider both macrostructural and microstructural PT asymmetries, as well as microstructural asymmetries in myelination (Andrulyte et al. 2024). In general, functional language lateralization is a complex trait that is likely determined by a variety of structural factors. Macrostructural asymmetries in the planum temporale explain little variance in individual functional language lateralization data. While microstructural asymmetries explain a larger amount of variance in functional lateralization, they are far from comprehensively predicting the functionally dominant hemisphere for language on their own. Thus, combining data from different structural modalities seems to be the most meaningful approach to maximize the explained variance in functional lateralization data for language.

## Conclusion and outlook

The findings described in the previous sections suggest that understanding the association between structural and functional asymmetries in the language system requires considering macro- and microstructural levels together. Moreover, it is important to recognize that language is a multifaceted construct: some language tasks may show associations, while others may rely on distinct processes and show little to no association. In addition to the language domain, functional hemispheric asymmetries have been observed in a variety of cognitive and executive domains, including visuospatial attention (Thiebaut de Schotten et al. 2011), manual praxis (Vingerhoets et al. 2012) emotional processing (Palomero-Gallagher and Amunts 2022), face processing (Rossion and Lochy 2022), body processing (Karlsson et al. 2022), inhibitory control (Aron et al. 2014; Villar-Rodríguez et al. 2024) and also for many motor behaviors such as handedness (Papadatou-Pastou et al. 2020), footedness (Packheiser et al. 2020) and cradling (Malatesta et al. 2019). The insights from this mini-review suggest that future studies on lateralized structure-function relationships in any of these cognitive systems or motor behaviors could benefit from taking microstructural asymmetries into account. Furthermore, the findings have significant implications for clinical neuroscience. Several neurodevelopmental and psychiatric disorders are associated with a higher prevalence of atypical hemispheric asymmetries (Mundorf et al. 2021a; Kong et al. 2022; Ocklenburg et al. 2024; Packheiser et al. 2025). PT asymmetry has been investigated in several conditions such as stuttering (Gough et al. 2018), epilepsy (Oh and Koh 2009; Pahs et al. 2013; Keller et al. 2018), schizophrenia (Petty et al. 1995; Pearlson 1997), bipolar disorder (Ratnanather et al. 2013), dyslexia (Heiervang et al. 2000), attention-deficit/hyperactivity disorder (ADHD) (Foster et al. 2002), and Alzheimer’s Disease (Kutová et al. 2018), among others. However, the findings of in studies were often inconsistent. For example, in schizophrenia one study found a striking reversal of planum temporal asymmetry (Petty et al. 1995), while others found no alterations (Rossi et al. 1994). A meta-analysis of PT asymmetry schizophrenia showed a significant reduction of PT asymmetry, but with a high heterogeneity of results (judging from the plots as no formal heterogeneity measures were provided in the meta-analysis) (Sommer et al. 2001). The findings of the present paper suggest that including microstructural measures of PT asymmetry may benefit clinical neuroscience research in hemispheric asymmetries in patients and may help in resolving such heterogeneous results.

## Data Availability

No datasets were generated or analysed during the current study.
